# The Impact of Implant–Abutment Connection on Clinical Outcomes and Microbial Colonization: A Narrative Review

**DOI:** 10.3390/ma13051131

**Published:** 2020-03-03

**Authors:** Dorina Lauritano, Giulia Moreo, Alberta Lucchese, Chiara Viganoni, Luisa Limongelli, Francesco Carinci

**Affiliations:** 1 Department of Medicine and Surgery, Centre of Neuroscience of Milan, University of Milano-Bicocca, 20126 Milan, Italy; moreo.giulia@gmail.com (G.M.); c.viganoni@campus.unimib.it (C.V.); 2 Multidisciplinary Department of Medical and Dental Specialties, University of Campania-Luigi Vanvitelli, 80138 Naples, Italy; alberta.lucchese@unicampania.it; 3 Interdisciplinary Department of Medicine, University of Bari, 70121 Bari, Italy; luisanna.limogelli@gmail.com; 4 Department of Morphology, Surgery and Experimental Medicine, University of Ferrara, 44121 Ferrara, Italy; crc@unife.it

**Keywords:** dental implants, dental implantation, peri-implantitis, periodontics, immediate dental implant loading, dental implant-abutment design, dental abutments

## Abstract

Introduction: Osseointegration are often suffering from oral conditions, especially, the micro gap at the implant–abutment connection represents a site for bacterial plaque aggregation, leading to increased inflammatory cells and causing peri-implantitis. Aim: The aim of this narrative review was to describe the different kinds of implant–abutment connections and their ability to reduce bacterial leakage and thus prevent peri-implantitis. Materials and methods: The following databases were consulted: Pubmed, Scopus, Cochrane Library, and Research gate and a total of 528 articles were found. After reading the abstract and titles, 473 items were excluded. The remaining articles (n = 55) were assessed for full-text eligibility. Thirty-three studies were included in the review. Results and Conclusions: We selected 22 clinical trials and 11 reviews, examining a total sample of 2110 implants. From the review, it was clear that there exists a relationship between the implant–abutment interface and bacterial leakage. All the connections presented an amount of micro-gap and bacterial micro-leakage, though conical and mixed connection systems seemed to behave better. Moreover, both connections seemed to have a better load distribution and the mixed system also had anti-rotational properties which are very useful during the positioning of the prosthesis.

## 1. Introduction

Implants are devices widely used to rehabilitate the edentulous area. In order to say that the placement of an implant is successful, it must be osseointegrated. The properties of the implant significantly influence the functional and structural connection between this last and the living bone, since it depends also on the mechanical properties of the implant surface [1]. Most of the implants are composed by titanium or titanium alloy, because these materials present high corrosion resistance, strength, toughness, resilience, low density and low stiffness [2,3]. However, new nonmetal bulk materials have been introduced: for example, zirconia is widely used in implantology, due to its high aesthetic results [4]. The study by Yeo et al. [5] described how chemical and biological modifications of dental titanium implants surface may enhance their osseointegration process, increasing the surface biocompatibility and bioaffinity with the hard tissue and accelerating the bone response to the surface. Albrektsson proposed the following criteria for implant dentistry that is still widely used [6]:

- The absence of clinical mobility of the implants;
- The absence of subjective sensitivity, pain or discomfort;
- The absence of peri-implantitis;
- The absence of persistent radiolucency around the implants;
- Bone loss lower than 0.2 mm annually after the implant’s first year of service;
- According to these criteria, the minimum levels for success are a success rate of 85% at the end of a five-year observation period and 80% at the end of a 10-year period.

Is important to underline that an implant is completely successful only when it is loaded and there is no bone resorption around it.

Peri-implantitis is influenced by bacterial plaque accumulation at the level of implant-abutment-connection (IAC). Two-piece implants unavoidably present a micro-gap between the implant and therefore the abutment [7]. These spaces, once early colonized, may constitute a bacterial reservoir that would subsequently contaminate the implant’s surroundings and interfere with the peri-implant tissues health. The presence of a micro-gap, and thus a reservoir of bacteria, when in close reference to the bone, may have a task in bone loss [8]. The bacteria found at the IAC level are often both anaerobic and facultative anaerobic, counting on the features of the microhabitat. Additionally, patients in danger of periodontitis have a greater risk of peri-implantitis [9,10].

The inflammatory content could increase due to the adhesion and proliferation of bacteria on the biofilm around the IAC during soft tissue manipulation for prosthetic component installation. The potential colonization of the implant–abutment micro-gap is probably related to multifactorial conditions, i.e., the precision fit between the implant components, which is associated with the implant system design, the torque used to connect the components, and the repeated screw loosening and re-tightening [11]. The structure of the IAC could have an impact on the amount of microbial leakage between the implant connection and the abutment. In addition, the success of the implant rehabilitation is related to mechanical properties, such as correct loading. Occlusal overloading after prosthetic rehabilitation can result in increased stress in both the implant and the implant–abutment connection, as well as in the surrounding bone. The IAC design and fit, influence the loading of the implant–abutment system during the physiological movements of the jaws. If this load is oversized, the implant system can fail due to screw fracture and loss, implant fracture, or damage to the prosthesis. To avoid failure in the implant system, the implant should be placed correctly and the prosthesis should be designed to minimize the length of the lever. In addition, occlusion should be designed so that the loads are transferred along the implant axis to prevent an excessive concentration of stress at the IAC [7,10].

Nowadays, the most common implant–abutment connections are as follows: external hexagonal, internal hexagonal, conical, and mixed (tapered connection with a geometrically defined one).

### Objectives

The aim of this review was to describe, according to the most recent literature, the different kinds of implant-abutment connections and their ability to reduce bacterial leakage, thus playing an important role in peri-implantitis prevention.

## 2. Materials and Methods

### 2.1. Eligibility Criteria

The criteria used to perform the review were as follows (Table 1):

### 2.2. Search

Electronic research was conducted using different databases to find articles on the relationship between the implant–abutment connection and bacterial leakage. In the review process, we only included English articles published during the last five years, in order to provide an updated overview about this topic. The following key-words, combined with the Boolean term “AND”, were used: “implant-abutment connection”, “fixture-abutment connection”, “IAC and bacterial leakage”, “implant-abutment interface”, “platform switching”, “bacterial leakage”, and “implant position”.

### 2.3. Study Selection and Data Collection Process

**Figure 1:**
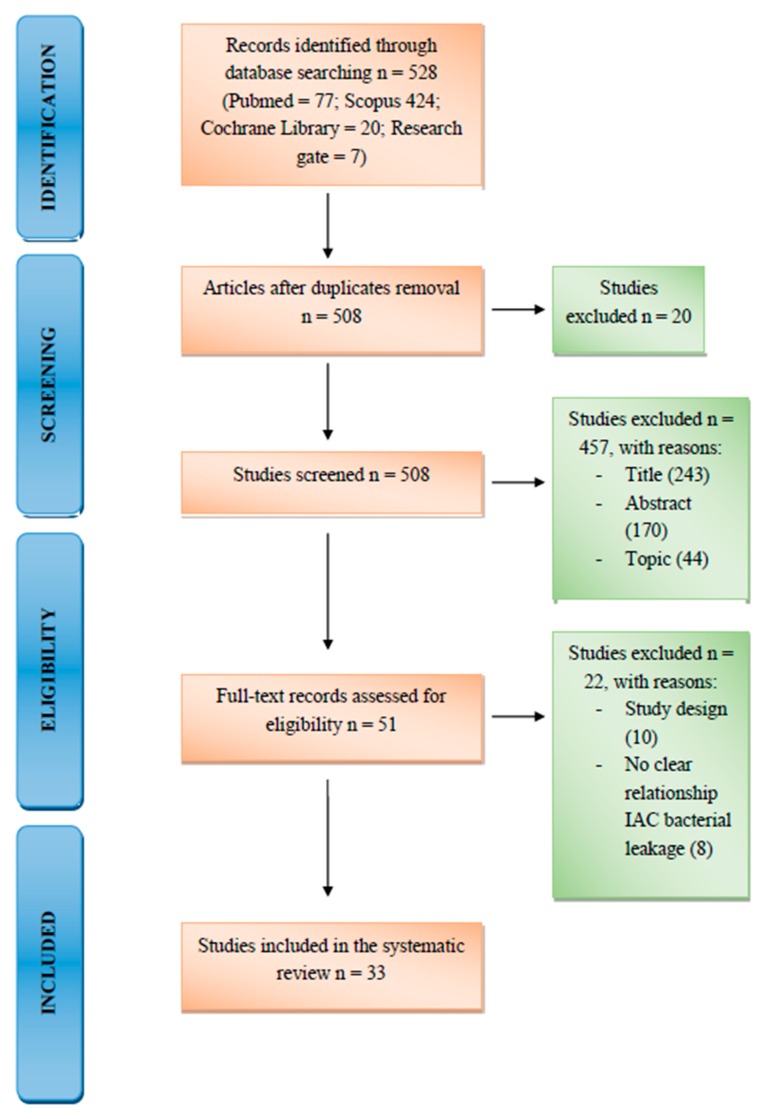
PRISMA Flow Diagram.

Eligibility assessment was performed by two masked independent reviewers, one of the authors of the review extracted the data from the included studies and a second author verified them. The disagreements between the reviewers were resolved with discussions between the two authors and if consent was not reached, the decision was taken by a third author. For each study was collected the following information: name of the author and year of publication, type of study (e.g., prospective cohort study, in vitro case-control), number of implant included in the single study, type of the implant, type of the implant-abutment connection, period of follow-up, exclusion criteria, confounding factors, limitations of the single study, type of the analysis performed for both in vivo and in vitro study (Table 2 and Table 3). The flow chart used for this study is shown in Figure 1. 

### 2.4. Risk of Bias

The four-stage approach of ROBIS tool (www.robis-tool.info) was used in order to assess the risk of bias of our systematic review. The evaluation process highlighted a low risk of bias: the eligibility criteria were predefined, unambiguous and appropriate for the review question; adequate terms and structure of the search strategy were used in order to find as many eligible studies as possible and articles were excluded only after abstract or full text screening. Furthermore, eligibility assessment was performed by two masked independent reviewers. However, restrictions regarding language and year of publication were imposed (only English studies published during the last five year were selected). The main risk of bias in our study derives from the fact that the included items took in consideration very different sizes of samples and different implant systems, produced by various companies. This condition made it difficult to perform a precise and statistically significant comparison between all the collected data.

## 3. Results

The following databases were consulted: Pubmed (n = 77), Scopus (n = 424), Cochrane Library (n = 20), and Research gate (n = 7) and we found a total of 528 articles. Duplicates were excluded (n = 20) and after reading the abstract and titles, we excluded the articles that were off topic (n = 457). The remaining articles (n = 55) were assessed for full-text eligibility: we excluded 10 articles because they were case reports and eight because there was no clear reference to the relationship between the IAC and bacterial leakage. A total of 33 studies were finally selected for this review. Table 2 and Table 3 summarize the characteristics of the 22 selected clinical trials. These data were added to the information, and conclusions drawn from the 11 reviews included in the research. Nine of the selected clinical studies were in vitro studies, the majority of which were case-control studies, where two or more different implant connections were compared. Regarding in vivo studies, four were case-control studies, three prospective clinical trials, two randomized clinical trials, and two retrospective trials. There was also one cross-sectional study and one prospective cohort study. Studies in which implants placed in both the mandible and the maxilla were considered. If the study was performed in vivo, the implants were loaded into the oral cavity, if the study was performed in vitro, the implants were inoculated with bacteria or subjected to cyclic loading. In both cases, through laboratory tests (e.g., PCR, culture exam) it was evaluated whether bacterial leakage was present at the implant-abutment connection level and which connection was able to reduce it to a minimum. If the study was performed in vivo, bone resorption was evaluated by periapical radiographs, if the study had been performed in vitro, the implants were subjected to cyclic loading and the possibility of detorque or screw and implant fracture was evaluated. The studies that has been taken into consideration for this narrative review are as follows: in vivo and in vitro studies, prospective and retrospective studies and other review. The minimum time of follow-up for these studies was: 48 hours if the study was performed in vitro and one year if it was an in vivo study.

All these studies took into consideration very different sizes of samples: Mencio et al., considered only 15 implants, while Cassetta et al., collected a sample of 748 implants. Overall, excluding data extrapolated from the analyzed literature reviews, our review examined a total sample of 2110 implants. The authors used different implant systems, produced by various companies, and this was one of the aspects that made it difficult to achieve a precise and statistically significant comparison between all the collected data. The use of different implant systems also involved the presence of different implant connections: nonetheless, it was possible to divide the various types of implants analyzed into four categories: single or double internal hexagonal connection, conical connection, mixed connection, and platform switching. Inclusion and exclusion criteria also varied a lot from study to study, but in general, it was possible to state that the main exclusion criteria reported were: alcohol abuse, smoking habit, systemic diseases (HIV infection, immunosuppression, diabetes, hematological diseases, neoplasms), oral mucosal disease close to the implant site, periodontitis, assumptions of drugs representing a contraindication to implant placement (e.g., bisphosphonates). Three studies, two conducted by Andreasi Bassi et al., in 2016 and one conducted in 2016 by Lopez, also included the presence of parafunctions, especially bruxism, in the exclusion criteria. Results of all these studies were collected at different times: in vitro studies used an average follow-up of 10.5 days, with a minimum of 48 h (Carinci et al., and Nayak et al.) and a maximum of one month in the study by Arshadi et al. In vivo studies instead had an average follow-up of about three years, with a maximum time of five years in the studies by Canullo et al., and Lopez et al., and a minimum of one year in the study by Mencio et al. However, the research reported by the included studies was conducted in different ways: most of the in vitro studies evaluated bacterial contamination of the implants using PCR after having left them, for a variable time, in solutions enriched with bacteria. On the other hand, in vivo studies evaluated different responses: bone loss, loss of attachment, implant stability, and radiographic aspects. The main limitations reported by the studies included: short period of loading, limited sample size, and use of periapical radiographs, which do not evaluate lingual and buccal bone loss. Data analysis was complex, as the various studies used not only different types of implants, but also different protocols. The decision to include both in vivo and in vitro studies derived from the desire to evaluate a global vision on the topic, which the literature has expressed over the last five years. Therefore, it was not possible to carry out a complete statistical evaluation or to extract specific data from the studies, as the differences were too marked, and the risk of bias was too high. However, what emerged from the sample of 2110 implants that we examined was that the conical and mixed form had a beneficial gap and bacterial micro-leakage, and a better response to the mechanical load, with excellent anti-rotational properties, compared to the external connection or the internal hexagonal connection. Results of individual studies are recorded in Table 4. 

Gherlone et al., demonstrated that, after the inoculation of an *Escherichia coli* suspension, the internal conical connection guarantee a significant reduction of microleakage at 96 h in comparison with the other control internal connections: only 30% of the implants with the conical connection were contaminated, unlike the controls group, whose percentage of contaminated implants was equal to 100%. 

With regard to the included articles that analyzed vertical bone resorption with periapical radiographs, the analysis shows that in two studies out of three (Sesma, 2016 and Cassetta, 2016), which analyze the platform switching connection, there is a reduction in peri-implant bone resorption. Sesma et al., recorded a vertical bone change equal to 0.40 ± 0.19 one year after functional loading, while in the study by Cassetta et al., the mean marginal bone remodeling was −0.56 mm. However, with regard to platform switching there are still discordant results. As Enkling, 2013 and Sesma, 2016 found in their studies the results obtained with platform switching and with standard connections are superimposable. Enkling et al., inserted two implants crestally in posterior mandible of 25 patients and, after three years of follow-up, data obtained showed that the mean radiographic peri-implant bone loss was 0.69 ± 0.43 mm in platform switching connections and 0.74 ± 0.57 mm in standard platform ones. 

Microbiological analysis were conducted within the cross-sectional study by Canullo et al., so as to gauge the bacterial microflora present inside the implant connections. Only 10% of the implants with conical connections showed positivity to red complex bacteria (*Porphyromonas gingivalis*), in comparison with double internal hexagon connections, which showed a positivity of 68%.

The in vitro study by Lauritano et al., showed that the new antimicrobial polysiloxane coating on the implant-abutment junction surface could inactivate the microbial species, avoiding them to penetrate the internal of the implants: the total amount of bacteria living in the internal part of the implants with respect to those living outside was 0.31% for *Porphyromonas gingivalis* and 0.32% for *Tannerella forsythia*. The total bacterial count average in screwed implant-abutment connection was 3.7 × 10^8^ and those in cemented implant-abutment connection was 2.1 × 10^8^, recording no statistically significant differences (*p* = 0.32). The pathogenic threshold of this latter group was overcame in the case of five bacteria (*Porphyromonas gingivalis*, *Tannerella forsythia*, *Treponema denticola*, *Prevotella intermedia*, *Campylobacter rectus*), while the bacterial colonization of peri-implant sulci was over for only one bacterium (*Prevotella intermedia*). This data demonstrated that screwed implant-abutment connections reduce the risk of peri-implantitis onset.

A comparison between three different internal conical connection designs (nano fix, uNiQo and Elisir implant systems by FMD, Rome, Italy), performed by Carinci et al., proved that uNiQo implant system provided the highest bacterial leakage decrease from the inside to the outside of the implant-abutment connection: the median percentage of bacteria living in the inner side of the implants was 1.4% for uNiQo, 1.9% for nano fix and 2.6 for Elisir.

## 4. Discussion

It’s well documented in literature that a micro gap between 1 to 49 μm occurs at the IAC with different implant systems [9]. Since IAC is typically located under the soft tissue of the gingiva, frequently close to the bone level, a serious aspect in averting the contamination of the peri-implant tissues is the control of bacterial leakage through it. Especially in patients with previous periodontal diseases, the depletion of pathogenic bacteria should be considered crucial because the presence of the typical bacterial species of periodontitis is reported also in peri-implantitis. Hence a pathogenic microflora is often related to a higher risk of peri-implantitis, as of periodontal diseases. While the “purple”, “yellow”, and “green” complexes are not associated to disease, the “orange” (*F. nucleatum*, *P. intermedia*, *M. micros*) and “red” complexes (*P. gingivalis*, *T. forsythia*, *T. denticola*) are disease-related. 

A. *actinomycetemcomitans* is also considered as being periodontopathogenic, although it is not included in any group [9,12,13,14,15].

It is known that peri-implantitis and the loss of osseointegration are due to bacterial leakage through the IAC, but also to other factors, i.e., different positions of the implant shoulder on the bone crest; implant diameter to better engage the cortical bone; implant design (conical or cylindrical), and what its coil, implant length, material abutments are made of (zirconia abutments are more likely to have microleakage than titanium abutments and these should be used only in cases where there was a very high demand for aesthetics [16]); surgical technique; and bone characteristics, such as density and thickness [11,17]. The main implant connections and their most important characteristics are listed below.

### 4.1. Internal Hexagonal Connection

The internal hexagonal connection (i.e., a part of the abutment is inserted in the body of the implant) is currently the most widely used among the two-piece implant systems. This is most likely due to the fact that this connection ensures proper abutment seating, anti-rotational engagement, resistance to lateral forces, and excellent aesthetic results. This connection is reported as being less favorable to the infiltration of fluids than the external connection. Higher stability under loading conditions has been reported for different internal connections compared to external connections, which, under functional loading, promotes micro-movements of the abutment and thus bacterial leakage. The performance of bridge rehabilitation was observed to be worse than that in single crown rehabilitations [7,18,19,20,21].

### 4.2. Conical

The cone connection has a unique design with an internal joint design between two conical structures. The internally tapered design creates a high propensity of parallelism between the two structures within the joint space, and it provides a significant amount of friction locks on the implant–abutment system [13]. Ceruso et al., pointed out that both conical and non-conical abutments provide enough resistance to maximal bending forces and fatigue loading. Still, conical abutments demonstrated superiority with regard to seal performance, micro-gap formation, torque maintenance, and abutment stability [22,23].

Loading forces on the prosthetic components may determine micro movements or IACs bending. This can lead to an enlargement of the micro-gap and a “pump effect” between the implant and the peri-implant tissues. Conical systems appear to control this inconvenience better than internal and external connections. According to He et al., the conical connection presents more resistance against formation of micro-gaps at the implant-abutment interface than the external hexagonal connection [24].

Another factor for long-term implant–abutment stability is the maintenance of torque value between the implant and abutment after tightening (the percentage of torque loss reported in the literature ranges between 16.1% and 25% [25]). Obviously, this can prevent abutment screw loosening or movement and also the micro-gap formation. All tested connection systems showed torque loss after initial tightening, particularly the external and internal hexagonal connection systems. Conical systems showed higher resistance to torque loss, and therefore, were less likely to increase the micro-gap and consequently decrease the probability of the occurrence of peri-implantitis. 

When using a conical connection, it’s important to evaluate the relationship between torque and gap at the IAC. The literature agrees that a progressive reduction in the gap between the implant and abutment occurs by increasing the torque. A tensile force within the screw stem is developed while applying the torque and it determines a compressive clamping force between the implant and abutment. Results demonstrated that a higher insertion torque in the cone connections reduced bacterial leakage. Therefore, torque values below 35 N/cm seem to reduce the contact between the abutment and the implant, resulting in weaker bacterial seals. 

Literature has also demonstrated the long-term superiority and predictability of the conical implant connection when submitted to axial and lateral loads in comparison to the internal and external hexagonal connection.

Finally, we can say that the conical implant connection system is more favorable insofar as the maintenance of the marginal bone is concerned [7,22,26,27,28,29,30,31].

According to Mencio et al., another factor that can influence bone resorption is the apico-occlusal position of the implant’s shoulder. Some authors recommended a subcrestal placement of two-piece implants, 2 to 3 mm below the cement–enamel junction of the neighboring teeth in aesthetic areas, in order to achieve an “acceptable emergence profile”. This apical positioning of the implant, however, results in an excessive length of the soft tissue dimension with concomitant persistent inflammation and possibly further loss of the supporting bone. However, only a few clinical studies have analyzed the influence of implant placement depth on the peri-implant to draw more detailed conclusions [12].

Once the implant is exposed, the clinician can decide between the cement-retained implant–abutment and screw-retained implant–abutment. The studies suggested that the first offers better results relating to fluid and bacterial permeability; and the second, instead, could be a reservoir of dangerous bacteria. According to the literature, the screwed implant–abutment connection showed 100% implants colonized by bacteria versus 20% in the cement implant–abutment connection. Nevertheless, the cement-retained implant–abutment may show a cement related peri-implantitis [13].

Nayak et al., have obtained a significant reduction of the bacterial leakage at the IACs level, using a specific gel or a particular rubber ring (O-ring). The gel showed to create a better seal thanks to its low viscosity, which allows it to flow easily throughout the IAC; whereas the rubber of the O-ring’s body can deteriorate over time, which may lead to bacterial spreading. The authors state that further evaluation is needed regarding this, as well as on the duration of the gel’s seal and the combination with an antimicrobial [32].

Arshad et al., have tried using an adhesive material in association with the abutment screw, and the results indicated that the abutment screws that were tightened by the adhesive material showed a significant increase in the removal torque value. The adhesive material can fill the space that exists in the abutment–implant junction surface in all of the commercial screw-retained implant systems. Considering what has been written in the article, using an adhesive material can reduce the micro leakage in the abutment–implant interface not only by filling the gap that exists in that interface, but also by improving the retention of the screw-retained abutments [33].

Siadat et al., found that the radiotracer technique was a precise and sensitive method for the evaluation of micro leakage in the IAC. Radioisotope offers a precise method, which is relatively inexpensive, reproducible, and provides the opportunity to quantitatively measure the micro- leakage [34].

Romanos et al., also tried to use a protocol of rinsing the abutment and the inner part of the implant with chlorhexidine, but it did not seem to have any effect on decontamination of the connections [35].

### 4.3. Mixed

The mixed connection is given by the union of a conic connection with a geometrically defined connection, for example, the conical plus octagonal morphology provides a cone, which terminates in an octagon. The cone gives the abutment remarkable stability; and the octagon instead has anti-rotational properties and precise positioning. With this connection, the principle of platform switching (the prosthetic platform is smaller than the implant platform) can be adopted. This method enables the distance between the margin of closure of the prosthesis and the crestal bone to be increased. In this way, it is possible to avoid bacterial leakage coming into direct contact with the bone margin, thereby helping in the prevention of bone resorption and peri-implantitis. Some authors suggest that the benefits of platform switching in a clinical setting could be advantageous, as long as limited edentulous spaces are present and narrow implants are not considered, especially in the aesthetic zone. However, from our bibliographic research, discordant results emerged on this topic: Enkling et al., found no statistically significant difference between the bone-level alteration with platform switching (−0.68 mm) and with the standard platform (−0.70 mm) [17]. Some authors suggest that longer follow-up (more than three years) should be done to clarify this aspect. Clinicians must keep in mind that peri-implant bone resorption is a multifactorial problem and other factors should be always analyzed: implant collar design, apico-coronal implant location, and soft tissue thickness. Special attention to the soft tissue thickness indicated a potentially key role in preventing peri-implant bone resorption: it was found that thin soft tissues (<2 mm) may prove peri-implant problems, despite the advised use of platform switching [10,11,36,37,38]. 

In conclusion, only a few studies have been noted to have a long follow-up period that evaluated the crestal bone level changes of conical connection implants with platform switching, and the influence of biologically relevant, anatomic and stress-related variables [12].

## 5. Conclusions

Up to now, no implant system or connection design has been able to provide a perfect sealing at the IAC. All the connections presented an amount of micro-gap and bacterial micro-leakage, though the conical, and mixed connection systems seemed to behave better (a gap of 10 μm was presented by the external connection implant, which was more than the Morse taper implants with a gap of 2–3 μm). 

In performing this literature review, there were limitations due to differences in the protocols under which the studies were performed. Not all the studies specified what kind of bacteria were analyzed, and some studies were performed in vivo whilst others were in vitro, and there was no homogeneity in the follow-up period.

## Figures and Tables

**Table 1:** Inclusion and exclusion criteria.

Inclusion Criteria	Exclusion Criteria
In vivo and in vitro studies	No clear reference to the relationship between implant-abutment-connection (IAC) and bacterial leakage
Prospective and retrospective studies	Case report because of limited clinical relevance
Systematic and narrative reviews	Studied with less than fifteen implants as sample size
	Less than 48 h of follow-up for in vitro studies
	Less than 14 days of follow-up for in vivo studies

**Table 2:** Characteristic of implants included in the study and follow-up periods.

Authors, Year	Type of Study	N° Implant Included	Type of Implant	Type of Connection	Follow Up
Gherlone, 2015	In vitro	80	Sweden and Martina	DAT connection—double action tight (internal connection)	96 h
Enkling, 2013	Rand. clinical trial	25	SICace, SIC-Invent AG	Internal hexagonical	3 years
Cassetta, 2015	Prospect. cohort study	748	Impladent	Platform-switching	3 years
Bassi, 2016	Prospect. clinical study	52	Elisir cylindrical, Elisir EVO conical	Internal hexagonical	4 years
Lopez, 2016	Retrospect. study	66	I-Fix	External Hex, Deep Conical, Internal Octagon, Internal Hex, Conical Connection	40 m
Garrana, 2016	In vitro	27	Southern Implants, Neodent, Straumann, Dentsply-Ankylos	External Hex, Deep Conical, Internal Octagon, Internal Hex, Conical Connection	7 days
Canullo, 2015	Cross-sectional study	40	BIOMET 3i, Sweden and Martina, ASTRA TECH Implant System	Double internal hexagon, internal hexagon, conical connection	5 years
Costa, 2017	In vitro case-control	24	Intraoss	External hexagonal	14 days
Arshad, 2017	In vitro case-control	20	Dentium	NR	1 m
Siadat, 2016	In vitro case-control	17	Nobel Biocare	NR	12 days
Romanos, 2014	Case-control	240	Dentsply Implants, Biomet 3i	Platform-switching, Morse tapered, internal polygonal butt-joint	2 years
Scarano, 2016	Clinical trial	146	Dental Tech	NR	NR
Ugurel, 2013	Case-control	64	Tasarimmed, Straumann, Biohorizons, Dentsply friadent	Screwless Morse taper	NR
Lauritano, 2017	In vitro case-control	40	Edierre Implants System	NR	96 h
Andreasi Bassi, 2016	Prospect. clinical study	133	EVO	Tapered connection	NR
Lopez, 2016	Retrospect. clinical study	215	Falappa Medical Devices	Conical connection	5 years
Carinci, 2016	In vitro case-control	17	Implant System FMD	FN, NQ, Eisir by FMD, Rome, Italy	48 h
Mencio, 2017	Rand. clinical trial	20	NR	Cemented implant-abutment and screwed implant-abutment connection	1 year
Gehrke, 2016	Case-control	40	NR	Conical internal connection	NR
Sesma, 2016	Case-control	36	Conexão (Conect AR)	Platform-switched and platform matched	15 m
Nayak, 2016	In vitro case-control	45	ADIN DentalImplant System	NR	48 h
Mencio, 2016	In vitro case-control	15	Winsix, BioSAF IN	NR	14 days

**Table 3:** Exclusion criteria, confounding factors and limitations of the included studies.

Summary of Exclusion Criteria	Summary of Confounding Factors	Summary of Limitation of the Studies	Type of Analysis (In Vivo Studies)	Type of Analysis (In Vitro Studies)
Alcohol Drug Smoking General health condition (liver, blood or kidney disease; immune-suppressed patients, corticosteroids therapy) Local tumors and ulcers Bruxism Pregnant women History of bisphosphonate Active periodontal or peri-implant pathology	Not clear best torque value Not all patients fulfill follow-up periods	Short follow-up Used periapical radiographs with no visibility of lingual and buccal bone Finding an appropriate adhesive material Sample size Lack of cyclic loading	Bone loss valued with periapical radiograph Sampling with paper point and following microbiological analysis (culture exam or PCR) *	Microbiological exam: PCR, culture exam, limulus, amoebocyte lysate * Cyclic loading and valuation of detorque, screw or implant fracture

NR = not reported. * Bacteria analyzed: Aggregatibacter actinomycetemcomitans, Porphyromonas gingivalis, Tannerella forsythia, Treponema denticola, Prevotella intermedia, Micromonas micros, Fusobacterium nucleatum, Campylobacter rectus, Eikenella corrodens and Candida albicans.

**Table 4:** Results of individual studies.

Study	Implant Connection	Outcome Measure	Results
Gherlone, 2015	Internal conical connection (ICC)	% of contaminated implants	ICC = 30% Controls = 100%
Sesma, 2016	Platform switching connection (PSC)	Vertical bone change one year after functional loading	0.40 ± 0.19
Cassetta, 2016	Platform switching connection (PSC)	Mean marginal bone remodeling	−0.56 mm
Enkling, 2013	Platform switching connection (PSC)	Mean radiographic peri-implant bone loss	PSC = 0.69 ± 0.43 mm Controls = 0.74 ± 0.57 mm
Cannullo, 2015	Conical connection (CC) vs. double internal hexagon connections (DIHC)	Positivity to red complex bacteria	CC = 10% DIHC = 68%
Lauritano, 2017	Antimicrobial polysiloxane coating on the implant-abutment junction (PCJ) vs cemented implant-abutment connection (CC)	Total bacterial count on average	PCJ = 3.7E + 08 CC = 2.1E + 08
Carinci, 2016	Internal conical connection design: nano fix vs. uNiQo vs. Elisir implant systems by FMD, Rome, Italy	Median percentage of bacteria living in the inner side of the implants	uNiQo = 1.4% nano fix = 1.9% Elisir = 2.6%
